# The Effect of Deuteration on the H_2_ Receptor Histamine Binding Profile: A Computational Insight into Modified Hydrogen Bonding Interactions

**DOI:** 10.3390/molecules25246017

**Published:** 2020-12-18

**Authors:** Lucija Hok, Janez Mavri, Robert Vianello

**Affiliations:** 1 Division of Organic Chemistry and Biochemistry, Ruđer Bošković Institute, HR-10000 Zagreb, Croatia; lucija.hok@irb.hr; 2 Laboratory for Computational Biochemistry and Drug Design, National Institute of Chemistry, SI-1001 Ljubljana, Slovenia; janez.mavri@ki.si

**Keywords:** deuteration, heavy drugs, histamine receptor, hydrogen bonding, receptor activation

## Abstract

We used a range of computational techniques to reveal an increased histamine affinity for its H_2_ receptor upon deuteration, which was interpreted through altered hydrogen bonding interactions within the receptor and the aqueous environment preceding the binding. Molecular docking identified the area between third and fifth transmembrane α-helices as the likely binding pocket for several histamine poses, with the most favorable binding energy of −7.4 kcal mol^−1^ closely matching the experimental value of −5.9 kcal mol^−1^. The subsequent molecular dynamics simulation and MM-GBSA analysis recognized Asp98 as the most dominant residue, accounting for 40% of the total binding energy, established through a persistent hydrogen bonding with the histamine −NH_3_^+^ group, the latter further held in place through the N–H∙∙∙O hydrogen bonding with Tyr250. Unlike earlier literature proposals, the important role of Thr190 is not evident in hydrogen bonds through its −OH group, but rather in the C–H∙∙∙π contacts with the imidazole ring, while its former moiety is constantly engaged in the hydrogen bonding with Asp186. Lastly, quantum-chemical calculations within the receptor cluster model and utilizing the empirical quantization of the ionizable X–H bonds (X = N, O, S), supported the deuteration-induced affinity increase, with the calculated difference in the binding free energy of −0.85 kcal mol^−1^, being in excellent agreement with an experimental value of −0.75 kcal mol^−1^, thus confirming the relevance of hydrogen bonding for the H_2_ receptor activation.

## 1. Introduction

Histamine is an important mediator and neurotransmitter that is involved in a broad spectrum of central and peripheral physiological as well as pathophysiological processes, such as allergies and inflammation. It exerts its specific effects by the activation of four receptor subtypes (H_1_R–H_4_R) [1]. Histamine receptors are 7-transmembrane receptors, which belong to the family of G-protein coupled receptors (GPCR), a very common target for a wide range of therapeutics used in modern pharmacotherapy, and differ in receptor distribution, ligand binding properties, signaling pathways and functions. Some estimates suggest that GPCRs encompass around 30% of the existing drug targets, while their therapeutic potential might be even larger [2,3].

The literature contains many studies on how GPCRs are activated and transmit their signals from the extracellular side to the G-protein coupling domain located on the intracellular side [4,5,6]. Instead, we have been interested in how different agonists and antagonists bind to the receptor binding site, and whether these processes are modulated upon non-selective deuteration, which would confirm the assumption that ligand binding is governed by hydrogen bonding interactions. Specifically, the topic of deuterium isotope effects is usually concerned with its impact on chemical reactions that are caused by substituting protium hydrogen (H) atoms with deuterium (D) in a molecule. These effects include changes in the rate of cleavage of covalent bonds to deuterium, or to an atom located adjacent to deuterium, in a reactant molecule. Alternatively, deuterium isotope effects on other, for example, noncovalent interactions between molecules are known to occur, but are generally considered to be insignificant, especially in biological experiments where deuterium substituted molecules are used as tracers. Nevertheless, replacing light hydrogen atoms with their heavier deuterium analogues, typically shortens the donor X–D bonds relative to the X–H bonds (X = heteroatom), as the X–D bonds are stronger, more compact and more stable to oxidative processes. Ultimately, this results in the elongation of the corresponding donor∙∙∙acceptor distance among heteroatoms, also known as the Ubbelohde effect [7], which affects the strength of the involved hydrogen bonds and can, therefore, produce modified affinities during the ligand–target recognition. Indeed, D has a 2-fold higher mass than H, leading to a reduced vibrational stretching frequency of the X–D bond compared to the X−H bond and, consequently, lower ground state energy. To further confirm that, Bordallo and co-workers recently performed a very accurate neutron diffraction study of the alanine zwitterion to show that deuteration reduces the electrostatic attraction in the acidic N–D bonds by 2.3% relative to the corresponding N–H bonds [8]. This results in the shortening of the N–D distances, as already noticed in various papers [9,10,11,12,13].

**Figure 1:**
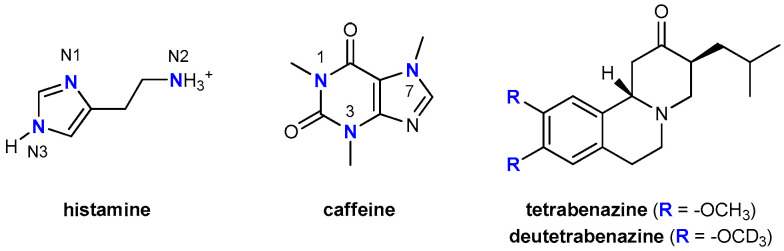
Chemical structures and atom labeling for the systems relevant to the discussion.

For many years, researchers have sought ways to incorporate deuterium into drug molecules in order to inhibit metabolic conversion into less active or inactive molecules [14,15], with the first such attempts being made nearly 60 years ago [16]. Because bonds to deuterium are stronger than those to hydrogen, early adopters tweaked molecules to better withstand the ravages of drug-metabolizing enzymes like cytochrome P450s. Deuterated drugs, they hoped, would have longer half-lives than their non-deuterated counterparts that would allow perhaps less frequent dosing and produce different metabolites. With this in mind, the focus was placed on drug fragments that were expected to be sites amenable to metabolic transformations, and typically involved chemical deuteration pertaining to heteroatom–CH_3_ groups (or other alkyl units) that were converted into heteroatom–CD_3_ alternatives. As an illustrative example, Falconnet [17], Brazier [18], and Cherrah [19,20] studied the binding of caffeine (Figure 1) to human serum albumin (HSA) by the equilibrium dialysis and demonstrated that the corresponding *K*_a_ values for caffeine, caffeine-1-CD_3_ and caffeine-1,3,7-(CD_3_)_3_ were not significantly different, while those for caffeine-3-CD_3_, caffeine-1,7-(CD_3_)_2_, and caffeine-3,7-(CD_3_)_2_ were considerably lower than that for caffeine, indicating that HSA had a reduced affinity for the deuterated compounds. On the other hand, very recently, the U.S. Food and Drug Administration granted market approval for the first deuterated drug molecule, deutetrabenazine (Figure 1), which is useful in treating chorea associated with Huntington’s disease [21]. Deutetrabenazine is a heavier analogue of the existing drug tetrabenazine, with two −OCH_3_ groups in the latter being replaced by a pair of −OCD_3_ groups, thereby altering the rate of metabolism to afford greater tolerability and an improved dosing regimen, thus an enhanced therapeutic potential was achieved. Still, in both of these instances, one can hardly argue that modified affinities came as a result of changed hydrogen bonding strengths, since methyl groups and their deuterated versions show poor hydrogen bonding abilities. Therefore, the observed effects likely originate in the modified dipole-dipole or dipole-charge interactions, which are generally weak. Knowing that hydrogen bonding interactions are significantly stronger than those mentioned, and that they typically dominate the ligand–target recognition [22], led us to offer some insight into the effect of deuteration on the ability of the H_2_ receptor to accommodate its endogenous agonist histamine, the latter particularly suitable to inspect alternations in the hydrogen bonding patterns and the accompanying affinities. Namely, histamine is a biogenic diamine (Figure 1), consisting of a free ethylamino group and an imidazole ring, thus involving three distinct sites able to either donate or accept hydrogen bonds, which makes it reasonable to expect that these particular interactions will predominantly govern its binding to the H_2_ receptor, as it was clearly demonstrated in the case of its hydration [23,24].

With this in mind, instead of utilizing chemical deuteration described earlier, in our preceding work [25], we have taken a different approach of introducing deuteration through the exchange mechanism by performing binding studies in pure D_2_O. In this way, we assured that all exchangeable hydrogen atoms, in both aqueous solution and within the H_2_ receptor will be replaced by deuterium, and that this will allow us to monitor how the hydrogen bonding interactions responsible for both the histamine hydration and its inclusion into the receptor binding site will be affected. Experiments were carried out on the H_2_ receptor present in cell membranes of cultured neonatal rat astrocytes, where we conducted the saturation and inhibition binding experiments using the antagonist ^3^H-tiotidine as a radiolabel, and histamine as a displacer of a bound radioligand. The results revealed a significant increase in the histamine affinity, as its pIC_50_ values (*p* < 0.05) changed from 7.25 ± 0.11 (control) to 7.80 ± 0.16 (D_2_O). Building on that, our subsequent work undertook the same approach for the binding of two agonists, 2-methylhistamine and 4-methylhistamine, and two antagonists, cimetidine and famotidine, and showed a notable affinity increase for 4-methylhistamine and a reduced one for 2-methylhistamine, while no change was observed for both antagonists [26]. This was interpreted in the context of the altered hydrogen bonding strength upon deuteration, which impacts ligand interactions with binding sites residues and solvent molecules preceding the binding. Our present work builds on the mentioned results [25,26], and considers the parent agonist histamine through a range of computational techniques, involving docking studies, classical molecular dynamics simulation, and quantum-chemical calculations within a large cluster model of the H_2_ receptor, in order to offer a more precise insight into the structural and electronic features of the studied ligand with the aim to provide the molecular interpretation to the observed binding differences. The outlined analysis is likely to contribute towards understanding the receptor activation, while the in silico discrimination between agonists and antagonists, based on the receptor structure, remains a distant ultimate goal.

## 2. Results and Discussion

As already mentioned, in our preceding work [25], we used ^3^H-tiotidine as a marker to label histamine H_2_ receptor binding sites on the cultured neonatal rat astrocytes, and histamine as an agonist to displace it, both in the control system and in deuterated environment. This resulted in a considerable deuteration-induced increase in the histamine affinity, as the measured pIC_50_ values (*p* < 0.05) went from 7.25 ± 0.11 (control) to 7.80 ± 0.16 (D_2_O). Although the relationship between IC_50_ and Δ*G*_BIND_ values is not so straightforward in absolute terms, their relative ratio is connected through the Cheng-Prusoff equation [27] and roughly translates to a difference of ΔΔ*G*_BIND_ = −0.75 kcal mol^−1^, which will be used in the rest of the text to evaluate the quality of computational results.

### 2.1. Docking Simulation

**Figure 2:**
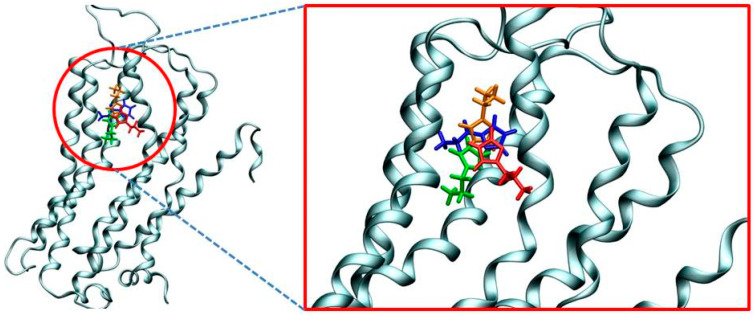
Overlap of four most favorable histamine binding poses within the H_2_ receptor as predicted by molecular docking that differ only in the ligand orientation. The computed binding free energies are −7.4 kcal mol^−1^ (blue), −7.1 kcal mol^−1^ (green), −6.8 kcal mol^−1^ (orange) and −6.7 kcal mol^−1^ (red).

To offer some initial insight into the binding of histamine into the H_2_ receptor, we employed several docking simulations with the aim of obtaining the relevant binding poses and the accompanying binding free energies, and use these as starting points for the subsequent molecular dynamics (MD) simulations. In doing so, we focused on the more stable N3–H (N^τ^) tautomer, which was docked into the homology structure of the H_2_ receptor. Interestingly, although the entire receptor surface was considered equally during the docking procedure, the obtained results reveal that the first four most favorable binding poses correspond to the identical position within the H_2_ receptor, and only differ in the conformation of the histamine ligand (Figure 2).

Apart from being positioned in the same binding pocket, a closer analysis of the predicted binding poses shows that all histamine molecules are located in the area between third and fifth transmembrane α-helices, in line with many earlier literature reports on the binding of H_2_ receptor ligands [28,29,30]. This provides some credence to the obtained results, which is further promoted by the calculated binding affinities. Namely, Figure 2 shows that the most favorable pose is associated with the binding energy of −7.4 kcal mol^−1^, which very well agrees with the experimental value of −5.9 kcal mol^−1^ obtained from the measured p*K*_i_ value of 4.3 [31]. Lastly, let us briefly mention that we have repeated the identical docking procedure for the less stable N1–H (N^π^) histamine tautomer, and the results showed an analogous placement within the H_2_ receptor and the identical binding energy of −7.4 kcal mol^−1^. Still, due to a described lower stability and the matching lower population of this tautomer relative to its N3–H analogue, N1–H tautomer was not considered further.

### 2.2. Molecular Dynamics Simulation of Histamine in Aqueous Solution

As already described, in aqueous solution, histamine exists almost exclusively (98%) as a monocation protonated at the free ethylamino group (Figure 1) and this protonation form was considered in a 20 Å-thick truncated octahedron simulation box, which involved 3.572 water molecules.

**Figure 3:**
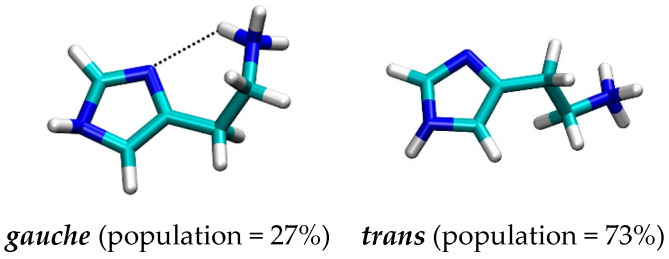
Representative conformations with their population of the histamine monocation in aqueous solution as obtained by the molecular dynamics simulation.

It turned out that histamine is a rather flexible molecule, but the clustering analysis of the obtained structures revealed a predominance of the two types of geometries (Figure 3), termed as *gauche*, in which there exists an intramolecular N–H∙∙∙∙∙N hydrogen bonding between the protonated amine (N2) as a donor and the imino nitrogen (N1) within the imidazole ring as an acceptor, and *trans*, which is elongated and where such a hydrogen bonding is absent. Interestingly, the results reveal around 73% dominance of the *trans* conformation, which is in an almost perfect agreement with around 80% predicted by other techniques [23,32,33,34,35]. It is worth mentioning that two useful geometric parameters, which characterize these two distinct orientations, and which will be used later in the analysis of the conformational preference of histamine within the receptor, are (i) distance between the relevant N1–N2 sites, and (ii) dihedral angle describing the rotation of the ethylamino group around the imidazole ring. In the representative *trans* geometry these are 4.54 Å and 158.8°, respectively, while in *gauche* these are reduced to 3.01 Å and 62.1°, in the same order. Their distribution during MD simulation (Figure S1) also indicates the preference of the *trans* conformation and further demonstrates the suitability of the described two structures as representative.

**Figure 4:**
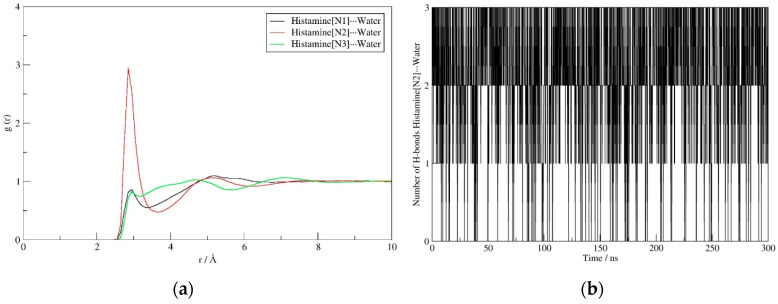
RDF displays describing the interaction of the solvent water molecules with N1 (black), N2 (red) and N3 (green) sites on histamine (**a**), and the evolution of the number of N2–H∙∙∙O(water) hydrogen bonds (**b**) during the molecular dynamics simulation in aqueous solution.

The interactions governing the hydration of histamine also reveal interesting trends. All three nitrogen sites (N1–N3) represent crucial locations to interact with water, with the corresponding RDF displays demonstrating an equal solvent ability to approach them (Figure 4a). Specifically, for all three positions, the predominant interactions are established at the N (histamine)∙∙∙O (water) distances of around 3 Å, which corresponds to rather strong hydrogen bonds in all cases. The interactions with both N-positions within the imidazole ring show identical patterns, thus indicating that N1 and N3 sites are participating as hydrogen bond acceptor and donor, respectively, with one water molecule in the first hydration shell. The latter is nicely evident in the average number of hydrogen bonding contacts, being 1.2 and 0.7 for N1 and N3, respectively. On the other hand, the interaction with the cationic N2 site is much more frequent, while around three times higher peak for N2 is only partially justified by the fact that the protonated amino group has three equivalent N2–H bonds that can potentially interact with three water molecules at the same time. As a matter of fact, Figure 4b advises that the actual number of hydrogen bonding contacts for this group is predominantly between 2 and 3, with an average value during simulation of 2.2, which is likely due to steric reasons and the exchangeability of individual solvent molecules. In addition, the shape of the RDF curve and a slightly lower distance for the peak maximum for N2 (2.8 Å relative to 2.9 Å for both N1 and N3), also suggests that its interactions with the solvent molecules are stronger than with the other two nitrogen sites. This notion is found in excellent agreement with our earlier report [24], where we utilized the Car-Parrinello molecular dynamics simulation scheme to delineate the experimental IR spectra of histamine in water, which showed a broad feature between 3350 and 2300 cm^−1^ including a mixed contribution from the ring N3–H and the aminoethyl N2–H stretching vibrations, to indicate that the ring amino group absorbs at higher frequencies than the remaining three amino N2–H protons, thus implying the latter forms stronger hydrogen bonding with the surrounding waters.

### 2.3. Molecular Dynamics Simulation within the H_2_ Receptor

Following the presented docking analysis, the obtained four most favorable docking positions, which differ in the orientation of the histamine ligand within the same binding pocket, were solvated in a 10 Å-thick truncated octahedron simulation box involving 18.850 water molecules, and submitted to the MD simulation for the production run of 300 ns. The validity of this approach is justified through the corresponding RMSD graphs, which reveal converged simulation (Figure S2). The obtained trajectories were analyzed by the MM-GBSA protocol in order to obtain the matching binding free energies, Δ*G*_BIND_. The mentioned four independent simulations gave Δ*G*_BIND_ values between −7.5 and −14.2 kcal mol^−1^, with the trajectory corresponding to the most exergonic binding being employed in further analysis. At this point it is worth to stress that the obtained Δ*G*_BIND_ values using this approach are somewhat overestimated in absolute terms. This is a known limitation of the MM-GBSA approach, as extensively discussed in a recent review by Homeyer and Gohlke [36], which also underlined its huge potential in predicting relative binding energies in the biomolecular complexes [36], which is how this approach is utilized here.

**Figure 5:**
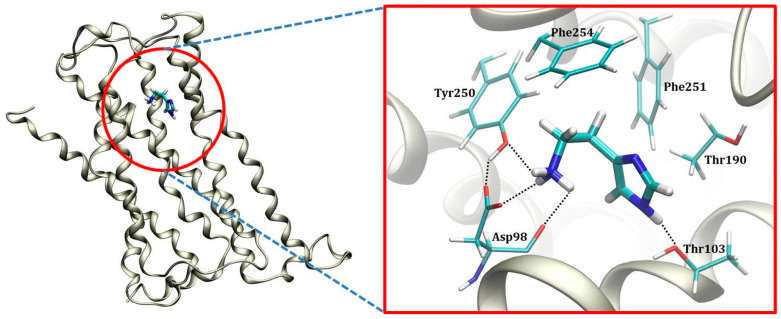
Representative position of the histamine monocation within the H_2_ receptor binding site as obtained from the molecular dynamics simulation.

The contribution of crucial binding residues is presented in Table 1, while a representative snapshot from the MD trajectory is shown in Figure 5. The specific residues considered for the analysis are those whose favorable contribution exceeds −0.06 kcal mol^−1^ and those with unfavorable contribution over +0.02 kcal mol^−1^, in total 18 residues each. It turns out that the most dominant interaction that histamine establishes within the H_2_ binding site is that with Asp98, which accounts for almost 40% of the binding energy, being a significant observation. It is established through charge-charge interactions among the protonated amino group on histamine and the anionic carboxylate side chain of Asp98, being highly persistent through the entire simulation, while typically involving N–H∙∙∙∙∙O hydrogen bond with only one of the carboxylic oxygen atoms (Figure S3). The reason for the latter is the fact that, besides Asp98, the protonated histamine −NH_3_^+^ group has the potential to interact with the –OH group on the nearby Tyr250, which is locked in the position through donating a hydrogen bond to the other carboxylic O-atom on Asp98 (Figure 5). The mentioned histamine∙∙∙Tyr250 interaction is also persistent during simulation (Figure S4, top), evident in a favorable Tyr250 contribution of −0.83 kcal mol^−1^, while Tyr250∙∙∙Asp98 hydrogen bonding is absent in the beginning, but, once formed, it remains stable during the second half of the trajectory (Figure S4, bottom).

The imidazole ring is less prone to hydrogen bonding interactions. This holds in particular for its imino N1 nitrogen, for which no particular interactions are observed at all during the entire simulation. In contrast, its amino N3–H group is found in the vicinity of two threonine residues, Thr103 and Thr190. Although N3–H∙∙∙∙∙O hydrogen bonding interactions with the former are more significant in this respect (Figure S5), thus the higher individual contribution of Thr103 over Thr190 (Table 1), both of these are much less frequent and clearly weaker than those with the −NH_3_^+^ group. Still, the individual contribution of Thr190 is quite notable, −0.84 kcal mol^−1^, not being a consequence of the mentioned hydrogen bonds with the ligand, but rather notable C–H∙∙∙∙∙π interactions with its imidazole ring (Figure 5). It is very important to underline that this binding pattern is different from the model by Birdsall and co-workers [37] proposed on the basis of the site-directed mutagenesis carried out by Gantz and co-workers [38] that suggested Thr190 to bind histamine on its N1 imino nitrogen through the O–H∙∙∙∙∙N1 hydrogen bonding. Yet, a closer inspection into the environment around Thr190 shows its –OH group forms a persistent and stable hydrogen bonding with a more distant backbone carbonyl group of Asp186 during 92% of the simulation time (Figure S6) with the average O∙∙∙∙∙O distance of 2.76 Å, thus ruling out Birdsall’s proposal as highly unlikely. To further strengthen this conclusion, we have looked at all other MD trajectories to find an alternative histamine orientation linked with a higher individual Thr190 contribution, which would likely indicate a potential hydrogen bonding connection with the ligand. However, in the case when this was as much as −1.48 kcal mol^−1^ for Thr190, this involved a completely different histamine orientation (Figure S7), yet having as much as 3.3 kcal mol^−1^ less exergonic overall binding free energy (−10.9 kcal mol^−1^), thus being much less relevant. There, the protonated histamine −NH_3_^+^ group approaches Asp186 through hydrogen bonding interactions, which makes the latter residue the most relevant for the binding, with an individual contribution of −3.19 kcal mol^−1^. This is, then, followed by Thr190, which, even in this case, does not form hydrogen bonding contacts with histamine, neither with the neighboring −NH_3_^+^ group, let alone with its imidazole ring, but rather again interacts through the already described C–H∙∙∙∙∙π interactions. Let us also mention that such a changed histamine position diminishes the importance of Asp98 and Tyr250 (Figure S7), making the former even disfavoring the binding with the contribution of +0.02 kcal mol^−1^, thus again confirming the insignificance of such ligand binding poses.

**Figure 6:**
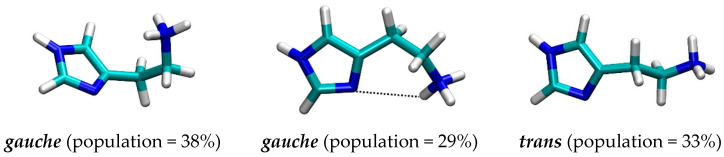
Representative conformations along with their populations of the histamine monocation at the H_2_ receptor binding site as revealed by molecular dynamics simulation.

Beside the mentioned receptor residues, the rest of the binding pocket is significantly hydrophobic, consisting mostly of aliphatic and aromatic side chains (Figure 5, Table 1). Nevertheless, this still allows histamine to establish a range of additional favorable contacts, including (i) its imidazole ring undergoing the T-shaped π–π stacking interactions with Phe251, and (ii) its ethyl moiety utilizing the C–H∙∙∙∙∙π interactions with Phe254 (Figure 5). Both of these contacts are rather strong and particularly important, making Phe254 and Phe251 the third and fifth most dominant residues for the histamine binding, with individual contributions reaching −1.60 and −0.97 kcal mol^−1^, respectively (Table 1). This confirms the hydrophobic nature of the H_2_ receptor binding site, further prompted by a significant contribution of Val99 of −1.78 kcal mol^−1^. Still, likely the most profound evidence for the hydrophobic character of the H_2_ binding pocket is the conformation of histamine during the binding. As already described, in polar hydrophilic environments, such as the aqueous solution, histamine predominantly assumes the elongated *trans* conformation, which disfavors the intramolecular N2–H∙∙∙∙∙N1 hydrogen bonding and exposes the protonated −NH_3_^+^ group for the interactions with the solvent. In contrast, the increased environment hydrophobicity starts favoring the *gauche* conformation, where the mentioned hydrogen bonding occurs and is allowed by the flexibility of the ethyl linkage. As an illustrative example, in the gas phase, the *gauche* conformer is by as much as 14.8 kcal mol^−1^ more stable [23], and clearly dominates in this paradigmatic hydrophobic media. Along these lines, the conformational preference of the bound histamine reveals interesting trends (Figure 6). The clustering analysis of histamine conformations while inside the H_2_ receptor shows that, for two thirds of the simulation time, histamine assumes *gauche* conformations, with a mix of structures with and without the N2–H∙∙∙∙∙N1 hydrogen bonding, while only one third of structures is found in a typical *trans* conformation, thus confirming the hydrophobicity of the H_2_ receptor interior. Such a distribution of histamine conformations is further evident in the evolution of the corresponding N1–N2 distances and dihedral angles describing the rotation of the ethyl chain (Figure S8), which primarily assume values that support the predominance of the *gauche* orientations.

### 2.4. Quantum-Chemical Calculations

In order to computationally evaluate the effect of deuteration on the binding of histamine to the H_2_ receptor, we have undertaken a series of quantum-chemical calculations at the M06–2X/6–31+G(d) level of theory, employing an implicit quantization of the acidic N–H, O–H and S–H bonds, and utilizing the implicit SMD solvation model with the dielectric constants of ε = 78.4 for aqueous solution and ε = 4.0 for the receptor interior, as already described. Also, we must note that, before reaching the receptor interior, the ligand is present in the aqueous solution, thus our approach is based on evaluating how deuteration affects both environments individually. This results in the separation of the deuteration-induced change in the overall ligand binding affinity, ΔΔ*E*_BIND_, into the contribution arising from the energy of hydration, ΔΔ*E*_HYDR_, and the energy of the interaction with receptor, ΔΔ*E*_INTER_, according to the following equation:

ΔΔ*E*_BIND_(H → D) = ΔΔ*E*_HYDR_(H → D) − ΔΔ*E*_INTER_(H → D)

In other words, the introduction of deuterium changes not only the geometric parameters of the matching deuterated bonds, but also the energies of the hydrogen bonding interactions in which these bonds participate. It is a fact that deuteration typically reduces the strength of the hydrogen bond, particularly if only one such interaction is considered. Yet, in this case, we are concerned with multiple hydrogen bonds that determine both the hydration and the interaction with the receptor, and since the overall effect (ΔΔ*E*_BIND_) is a difference between these two quantities, it can, at the end, be either positive or negative, depending on the ligand. In line with that, our earlier report on the same receptor showed that deuteration increased the binding of 4-methylhistamine and gave a reduced affinity for 2-methylhistamine, while offered no change for antagonists cimetidine and famotidine [26]. With this in mind, we have extracted the relevant snapshots from the MD simulation in water and H_2_ receptor, both with and without histamine, and truncated the geometries to clusters involving 36 molecules of water, and receptor residues 98–103, 186–190 and 250–254, respectively. These were submitted to an unconstrained optimization of all geometric parameters, corresponding to the situation with lighter H-nuclei, to be followed by manually shortening by 2.3% and constraining all acidic N–H, O–H and S–H bonds that mirrors deuterated analogues. For histamine, the latter involved all four N–H bonds, three within the protonated N2 amino group and one within the ring N3–H moiety, while for the water molecules this included all O–H bonds. For the receptor fragments, this pertained shortening the O–H bonds in Thr103, Thr190, Tyr250 and Thr252, and the S–H bond in Cys102. This choice is supported by knowing that threonine, having the least acidic side chain moiety of all three considered residues, spontaneously exchanges all of its −OH protons in D_2_O [39], thus justifying the same approach for more acidic tyrosine and cysteine residues.

**Figure 7:**
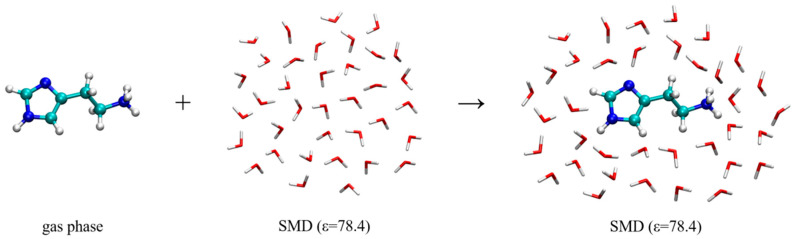
Computational scheme to calculate the hydration energy of the histamine monocation in the aqueous solution, Δ*E*_HYDR_. The selection of dielectric constants is specified in round brackets.

The hydration energy, Δ*E*_HYDR_, is calculated using a reaction scheme depicted in Figure 7 and the obtained values are given in Table 2. This approach relies on transferring histamine from the gas phase into the aqueous solution and forming a hydrated solute-solvent complex. In water, the hydration energy is calculated as −71.63 kcal mol^−1^, indicating that the histamine monocation is well solvated and stabilized in water. This value is reduced in D_2_O, as a result of modified hydrogen bonding interactions and their strength following deuteration, and assumes −71.20 kcal mol^−1^, a small effect of only 0.43 kcal mol^−1^ in favor of H_2_O. This implies that the hydration, on its own, works in the direction of promoting the binding of a deuterated system to the receptor.

**Figure 8:**
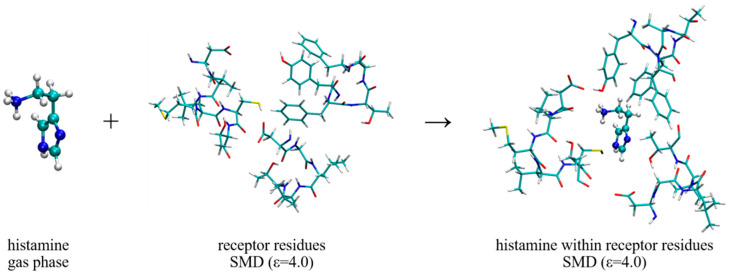
Computational scheme to estimate the interaction energy of the histamine monocation with the H_2_ receptor, Δ*E*_INTER_. The selection of dielectric constants is specified in round brackets.

On the other hand, the interaction energies with the receptor, Δ*E*_INTER_, are estimated through the scheme shown in Figure 8, which considers placing a ligand from the gas phase into the cluster model of the receptor binding site. We have to mention that, despite considering only a truncated receptor model and approximating the rest of its structure with the dielectric constant of ε = 4.0, during the geometry optimization, the structure of histamine and its protonated −NH_3_^+^ group remained as such, although it is positioned in the direct interaction with the –COO^−^ group from Asp98. In other words, we did not observe a spontaneous histamine∙∙∙Asp98 proton transfer, which could have occurred due to a limited account of the electrostatic environment that disfavors charge separation. Instead, both the structure and the position of histamine within the binding site remained as described during the MD simulation, which justifies our model and the selection of the most important residues for the cluster-continuum approach. With this in mind, it is important to notice that the interaction energies, Δ*E*_INTER_, are consistently higher than Δ*E*_HYDR_, which confirms histamine ability to leave the aqueous solution and enter the receptor. In water, this assumes Δ*E*_INTER_ = −82.31 kcal mol^−1^, which is, interestingly, further increased by 0.42 kcal mol^−1^ to Δ*E*_INTER_ = −82.73 kcal mol^−1^ upon deuteration. This already indicates that deuterated histamine is better accommodated within the receptor, but the precise magnitude of the resulting effect is interplay between this interaction and histamine placement in the aqueous solution preceding the binding.

Combining the mentioned hydration and interaction energies and their differences, one arrives to the overall change in the binding energy for the receptor-ligand recognition, which assumes ΔΔ*E*_BIND,calc_ = −0.85 kcal mol^−1^, being in excellent agreement with the experimentally determined value of −0.75 kcal mol^−1^ [25], thus confirming an increased histamine affinity following deuteration. Both the calculated and computed values indicate around 3–4 times higher histamine affinity following deuteration, which is an interesting observation bearing some pharmacological relevance. The agreement between these sets of data is very impressive, particularly given the simplicity of the computational model used for the implicit deuteration conducted on only a small, but carefully selected part of the receptor molecule, which validates the employed methodology and allows its use in other biological systems as well.

In concluding this section, let us emphasize that receptor activation is a highly complex and dynamic process associated with large conformational changes between receptor states. These are difficult to investigate experimentally, while, at the same time, occurring on time scales that are inaccessible for direct molecular simulations. Still, the results presented here provide convincing support that hydrogen bonding interactions are involved in the receptor activation and firmly advise that deuteration, as the simplest possible structural modification, can have a significant impact for the ligand affinity. This opens the door for the development of perdeuterated drugs, which could have different, yet in some instances more favorable clinical profiles to already marketed substances.

## 3. Computational Details

A homology structure of the H_2_ receptor was developed earlier [25], which revealed a good agreement with other models reported in the literature [40,41], and was employed here throughout the entire work. The structure of histamine contains an imidazole ring and an aminoethyl side chain, both of which have the ability of accepting a proton, if the medium is acidic enough. According to its p*K*_a_ values, 6.0 for the imidazole nitrogen and 9.7 for the aliphatic amino group [42], at physiological pH of 7.4, histamine is predominantly a monocation (96%) protonated at the free amino group. Also, its imidazole ring can exist in two tautomeric forms, 1*H*-imidazole and 3*H*-imidazole (Figure 1), denoted as N^π^–H and N^τ^–H, respectively, with plenty of experimental and computational evidence in favor of the latter as the predominant structure in the aqueous solution [23,32,33,34,35]. With all this in mind, the structure of the histamine N^τ^–monocation was considered in all simulations.

### 3.1. Docking Analysis

The structure of the histamine monocation was optimized with the Gaussian 16 software [43] employing the M06–2X DFT functional with the 6–31+G(d) basis set. To account for the effect of the aqueous solution, during the geometry optimization we included the implicit SMD polarizable continuum model [44] with all parameters for pure water. The molecular docking studies have been done with SwissDock [45], a web server for docking of small molecules on the target proteins based on the EADock DSS engine, taking into account the entire protein surface as potential binding sites for the investigated ligands. Both the preparation of the H_2_ receptor structure and the visualization of results were performed using the UCSF Chimera program (version 1.14) [46].

### 3.2. Molecular Dynamics Simulation

Several best binding poses of histamine within the H_2_ receptor, elucidated through the preceding docking analysis, were used for subsequent molecular dynamics simulations. To parametrize histamine, RESP charges were calculated at the HF/6–31G(d) level of theory in Gaussian 16 program [43] to be consistent with the employed GAFF force field, while the protein was modeled using the AMBER ff14SB force field. Such a complex was then solvated in a truncated octahedral box of TIP3P water molecules spanning a 10 Å-thick buffer, neutralized by 12 Cl^–^ anions, and submitted to the geometry optimization in the AMBER16 program package [47], employing periodic boundary conditions in all directions. An analogous setup, involving a 20 Å-thick buffer of water molecules around isolated histamine monocation, joined by the Cl^−^ counterion, whose position was fixed at the border of the simulation box by a force constant of 30 kcal mol^−1^ and a position restrain between 19–21 Å from histamine, was utilized for the MD simulation pertaining to the aqueous solution. Both approaches were identically repeated to setup analogous simulations concerning the receptor and the aqueous solution without histamine and its counterion. In all instances, optimized systems were gradually heated from 0 to 300 K and equilibrated during 30 ps using NVT conditions, followed by productive and unconstrained MD simulation of 300 ns, employing a time step of 2 fs at a constant pressure (1 atm) and temperature (300 K), the latter held constant using a Langevin thermostat with a collision frequency of 1 ps^−1^. The long-range electrostatic interactions were calculated employing the Particle Mesh Ewald method [48], and were updated in every second step, while the nonbonded interactions were truncated at 11.0 Å.

Histamine binding free energies, Δ*G*_BIND_, within the H_2_ binding pocket were calculated using the established MM-GBSA protocol [49,50] available in AmberTools16 [47], and in line with our earlier reports [51,52]. MM-GBSA is widely used for calculating the binding free energies from snapshots of the MD trajectory with an estimated standard error of 1–3 kcal mol^−1^ [49]. For that purpose, 3000 snapshots collected from the last 30 ns of the corresponding MD trajectories were utilized. The calculated MM-GBSA binding free energies were decomposed into a specific residue contribution on a *per-residue* basis according to the established procedure [53,54]. This protocol evaluates contributions to Δ*G*_BIND_ arising from each amino acid residue and identifies the nature of the energy change in terms of the interaction and solvation energies or entropic contributions.

### 3.3. Quantum-Chemical Calculations

Following the MD analysis, which identified residues dominating the histamine binding, we took a representative snapshot and extracted positions of the bound histamine and the surrounding residues 98–103, 186–190 and 250–254. The same residues were pulled out from the H_2_ receptor MD simulation without histamine. In this way, the cluster representation of the receptor binding site consisted of the following residues Asp98, Val99, Met100, Leu101, Cys102, Thr103, Asp186, Gly187, Leu188, Val189, Thr190, Tyr250, Phe251, Thr252, Ala253 and Phe254, which were considered in their typical protonation forms according to the PROPKA 3.1 analysis [55] carried out on the entire homology structure. From the MD simulation in water, we extracted the position of the nearest 36 water molecules within 4 Å from histamine, which allowed for a spherical solvent layer involving histamine first solvation shell. An analogous cluster with the same number of waters was taken out from the MD simulation of a plain aqueous solution. In this way, we obtained the starting geometries for the quantum-chemical calculations involving the H_2_ receptor and the aqueous solution, both with and without histamine. These were submitted to a full geometry optimization at the M06–2X/6–31+G(d) level in Gaussian 16 [43]. Total molecular electronic energies were extracted without thermal corrections, so the results reported here correspond to differences in electronic energies. The effect of the rest of the receptor environment was considered through the implicit SMD solvation using a dielectric constant of ε = 4.0, as suggested by Himo and co-workers [56], and a dielectric constant of ε = 78.4 for the aqueous solution, in line with our previous reports [25,26,51]. In addition, such a truncated cluster-continuum model of the entire protein turned out to be very useful in rationalizing various aspects of the catalytic activity [57], selectivity [58] and inhibition [51] of the monoamine oxidase family of enzymes, and is broadly used by different groups to describe various biological phenomena [59,60,61,62,63], which justifies its use here.

Lastly, although the literature presents a number of methods for the quantization of nuclear motion, relevant for studying the H/D isotope substitution, these are limited to only a few degrees of freedom. Yet, these are not applicable here, since we have many critical protons directly involved in the H_2_ receptor-ligand recognition and water hydration. As such, we employed an approximate empirical treatment of the nuclear quantum effects based on the mentioned experimental work by Bordallo and co-workers [8], which showed that deuteration reduces the electrostatic attraction in the acidic N–D bonds by 2.3% relative to the matching N–H bonds. With this in mind, we imposed the empirical quantization in the following way. Initially, all systems were fully optimized, thus mirroring the case with lighter H nuclei. After that, all acidic N–H, O–H and S–H bonds were shortened by 2.3% and kept frozen during the optimization of other geometric parameters, thus corresponding to heavier D nuclei, in accordance with our earlier reports [25,26].

## 4. Conclusions

This study relied on a range of computational techniques to demonstrate the significance of the hydrogen bonding and other non-covalent interactions for the binding of histamine to its H_2_ receptor, and evaluated how these are affected by deuteration. Molecular docking analysis determined histamine binding poses on the homology model of the H_2_ receptor, while molecular dynamics simulation underlined crucial residues governing the binding. This recognized Asp98 as the most dominant residue, accounting for 40% of the total binding energy, further held in place by Tyr250, which donates hydrogen bonding to Asp98 and accepts it from the histamine −NH_3_^+^ group. In contrast to earlier literature reports, we showed that the significant role of Thr190 is not in the −OH hydrogen bonds, but rather in the C–H∙∙∙π contacts with the imidazole ring, while the former is persistently involved in the hydrogen bonding with a more distant Asp186. The rest of the binding pocket is hydrophobic, allowing for a range of favorable contacts with Phe254, Phe251 and Val99, but also evident in a clear predominance for the *gauche* histamine conformation within the receptor, unlike the aqueous solution where it is *trans*. Molecular dynamics simulation in the aqueous solution revealed that the first histamine solvation shell involves five water molecules at all three nitrogen sites, yet the interaction with its −NH_3_^+^ groups mostly does not occur with three water molecules at the same time, but is linked with an average of 2.2 such contacts during the entire simulation.

Following molecular dynamics simulation, which identified receptor residues crucial for the binding and a representative cluster of 36 water molecules in the aqueous solution, quantum-chemical calculations at the M06–2X/6–31+G(d) level utilized the empirical quantization of the acidic X–H bonds (X = N, O, S) to support the increased histamine affinity upon deuteration. The overall binding was separated in two contributions, that from the interaction with the receptor and the one arising from the interaction with the solvent preceding the binding, which were both modeled through a cluster-continuum approach utilizing the implicit SMD solvation with the dielectric constants of ε = 4.0 for the receptor environment, and ε = 78.4 for the aqueous solution. The used computational setup gave the calculated difference in the binding free energy of −0.85 kcal mol^−1^, being in excellent agreement with the measured value of −0.75 kcal mol^−1^, thus confirming the relevance of hydrogen bonds for the receptor activation.

The results of this study highlight the importance of deuteration for the development of new drugs, as the selective replacement of exchangeable hydrogen atoms with deuterium can increase the duration of action due to their slower decomposition [64,65]. In addition, this can result in different, yet in some instances more beneficial clinical profiles to already marketed solutions, and further progress in this area is highly recommended. Finally, we are convinced that advanced molecular simulations of entire receptors with the inclusion of experimental data will finally lead to a methodology that will be able to discriminate between GPCR agonist and antagonists, which is currently limited to QSAR applications [66].

## Figures and Tables

**Table 1:** MM-GBSA binding free energy (Δ*G*_BIND_) from the molecular dynamics trajectory and its decomposition on a *per-residue* basis (in kcal mol^−1^). Residues are selected to list all of those with favorable contributions over –0.06 kcal mol^−1^ and unfavorable contributions over +0.02 kcal mol^−1^.

Histamine			
Δ*G*_BIND_ = −14.2 kcal mol^−1^			
Favorable Contributions		Unfavorable Contributions	
Asp98	−5.45	Leu193	+0.02
Val99	−1.78	Gly157	+0.02
Phe254	−1.60	Lys166	+0.02
Thr103	−1.29	Lys83	+0.02
Phe251	−0.97	Val273	+0.02
Thr190	−0.84	Asn108	+0.02
Tyr250	−0.83	Lys88	+0.02
Gly187	−0.64	Tyr192	+0.03
Asp186	−0.64	Arg260	+0.03
Met100	−0.20	Ala253	+0.05
Glu270	−0.16	Ser153	+0.05
Cys102	−0.14	Arg161	+0.06
Glu267	−0.11	Lys173	+0.11
Val178	−0.10	Gln177	+0.11
Tyr94	−0.08	Gly183	+0.13
Val189	−0.08	Arg257	+0.15
Val185	−0.07	Leu97	+0.19
Val255	−0.06	Lys175	+0.29

**Table 2:** Calculated deuteration-induced changes in the hydration energy (Δ*E*_HYDR_), H_2_ receptor interaction energy (Δ*E*_INTER_), and the overall receptor binding energy (Δ*E*_BIND,calc_) as obtained by the (SMD)/M06–2X/6–31+G(d) model (in kcal mol^−1^), the latter compared with the experimentally determined value (Δ*E*_BIND,exp_) from ref. [25].

Ligand	In H_2_O			In D_2_O				
	Δ*E*_HYDR_	Δ*E*_INTER_	Δ*E*_BIND_	Δ*E*_HYDR_	Δ*E*_INTER_	Δ*E*_BIND_	ΔΔ*E*_BIND,calc_	ΔΔ*E*_BIND,exp_
**Histamine**	−71.63	−82.31	−10.69	−71.20	−82.73	−11.54	−0.85	−0.75

## Supplementary Materials

The following are available online, Figures S1–S8 showing various analyses from the molecular dynamics simulation.

## References

[1] Walter M., Stark H. (2012). Histamine receptor subtypes: A century of rational drug design. Front. Biosci..

[2] Cong X., Topin J., Golebiowski J. (2017). Class A GPCRs: Structure, function, modeling and structure-based ligand design. Curr. Pharm. Des..

[3] Congreve M., Langmead C.J., Mason J.S., Marshall F.H. (2011). Progress in structure based drug design for G protein-coupled receptors. J. Med. Chem..

[4] Keshelava A., Solis G.P., Hersch M., Koval A., Kryuchkov M., Bergmann S., Katanaev V.L. (2018). High capacity in G protein-coupled receptor signaling. Nat. Commun..

[5] Mason J.S., Bortolato A., Congreve M., Marshall F.H. (2012). New insights from structural biology into the druggability of G protein-coupled receptors. Trends Pharmacol. Sci..

[6] Weis W.I., Kobilka B.K. (2018). The Molecular Basis of G Protein–Coupled Receptor Activation. Annu. Rev. Biochem..

[7] Ubbelohde A.R., Gallagher K.J. (1955). Acid-base effects in hydrogen bonds in crystals. Acta Cryst..

[8] De Souza J.M., Freire P.T.C., Bordallo H.N., Argyriou D.N. (2007). Structural isotopic effects in the smallest chiral amino acid: Observation of a structural phase transition in fully deuterated alanine. J. Phys. Chem. B.

[9] Shi C., Zhang X., Yu C.-H., Yao Y.-F., Zhang W. (2018). Geometric isotope effect of deuteration in a hydrogen-bonded host-guest crystal. Nat. Commun..

[10] Rivera-Rivera L.A., Wang Z., McElmurry B.A., Willaert F.F., Lucchese R.R., Bevan J.W., Suenram R.D., Lovas F.J. (2010). A ground state morphed intermolecular potential for the hydrogen bonded and van der Waals isomers in OC:HI and a prediction of an anomalous deuterium isotope effect. J. Chem. Phys..

[11] Mishra A.K., Murli C., Sharma S.M. (2008). High pressure Raman spectroscopic study of deuterated γ-glycine. J. Phys. Chem. B.

[12] Goncalves R.O., Freire P.T.C., Bordallo H.N., Lima J.A., Melo F.E.A., Mendes Filho J., Argyriou D.N., Lima R.J.C. (2009). High-pressure Raman spectra of deuterated L-alanine crystal. J. Raman Spectrosc..

[13] Smirnov S.N., Golubev N.S., Denisov G.S., Benedict H., Schah-Mohammedi P., Limbach H.-H. (1996). Hydrogen/deuterium isotope effects on the NMR chemical shifts and geometries of intermolecular low-barrier hydrogen-bonded complexes. J. Am. Chem. Soc..

[14] Yarnell A.T. (2009). Heavy-hydrogen drugs turn heads, again. Chem. Eng. News.

[15] Halford B. (2016). Deuterium switcheroo breathes life into old drugs. Chem. Eng. News.

[16] Belleau B., Burba J., Pindell M., Reiffenstein J. (1961). Effect of Deuterium Substitution in Sympathomimetic Amines on Adrenergic Responses. Science.

[17] Falconnet J.B., Brazier J.L., Desage M. (1986). Synthesis of seven deuteromethyl-caffeine analogues; observation of deuterium isotope effects on CMR analysis. J. Label. Compd. Radiopharm..

[18] Brazier J.L., Ribon B., Falconnet J.B., Cherrah Y., Benchekroun Y. (1987). Etude et utilisation des effets isotopiques en pharmacologie. Therapie.

[19] Cherrah Y., Falconnet J.B., Desage M., Brazier J.L., Zini R., Tillement J.P. (1987). Study of deuterium isotope effects on protein binding by gas chromatography/mass spectrometry. Caffeine and deuterated isotopomers. Biomed. Environ. Mass Spectrom..

[20] Cherrah Y., Zini R., Falconnet J.B., Desage M., Tillement J.P., Brazier J.L. (1988). Study of deutero-isotopomer-induced inhibition of caffeine and phenobarbitone binding to human serum albumin. Biochem. Pharmacol..

[21] Schmidt C. (2017). First deuterated drug approved. Nat. Biotechnol..

[22] Toth G., Bowers S.G., Truong A.P., Probst G. (2007). The Role and Significance of Unconventional Hydrogen Bonds in Small Molecule Recognition by Biological Receptors of Pharmaceutical Relevance. Curr. Pharm. Des..

[23] Vianello R., Mavri J. (2012). Microsolvation of the histamine monocation in aqueous solution: The effect on structure, hydrogen bonding ability and vibrational spectrum. New J. Chem..

[24] Stare J., Mavri J., Grdadolnik J., Zidar J., Maksić Z.B., Vianello R. (2011). Hydrogen bond dynamics of histamine monocation in aqueous solution: Car–Parrinello molecular dynamics and vibrational spectroscopy study. J. Phys. Chem. B.

[25] Kržan M., Vianello R., Maršavelski A., Repič M., Zakšek M., Kotnik K., Fijan E., Mavri J. (2016). The quantum nature of drug-receptor interactions: Deuteration changes binding affinities for histamine receptor ligands. PLoS ONE.

[26] Kržan M., Keuschler J., Mavri J., Vianello R. (2020). Relevance of Hydrogen Bonds for the Histamine H2 Receptor-Ligand Interactions: A Lesson from Deuteration. Biomolecules.

[27] Cheng H.C. (2001). The power issue: Determination of *K*_B_ or *K*_i_ from IC_50_—A closer look at the Cheng–Prusoff equation, the Schild plot and related power equations. J. Pharmacol. Toxicol. Methods.

[28] Singh V., Gohil N., Ramírez-García R. (2018). New insight into the control of peptic ulcer by targeting the histamine H_2_ receptor. J. Cell Biochem..

[29] Strasser A., Wittmann H.J., Hattori Y., Seifert R. (2017). Molecular Modelling Approaches for the Analysis of Histamine Receptors and Their Interaction with Ligands. Histamine and Histamine Receptors in Health and Disease. Handbook of Experimental Pharmacology.

[30] Sun X., Li Y., Li W., Xu Z., Tang Y. (2011). Computational investigation of interactions between human H_2_ receptor and its agonists. J. Mol. Graph. Model..

[31] Schreeb A., Łażewska D., Dove S., Buschauer A., Kieć-Kononiwcz K., Stark H., Stark H. (2013). Histamine H_4_ receptor ligands. Histamine H_4_ Receptor: A Novel Drug Target for Immunoregulation and Inflammation.

[32] Collado J.A., Tuñón I., Silla E., Ramírez F.J. (2000). Vibrational Dynamics of Histamine Monocation in Solution: An Experimental (FT-IR, FT-Raman) and Theoretical (SCRF-DFT) Study. J. Phys. Chem. A.

[33] Collado J.A., Ramírez F.J. (2000). Vibrational spectra and assignments of histamine dication in the solid state and in solution. J. Raman Spectrosc..

[34] Drozdzewski P., Kordon E. (2000). Isotope effects in the far-infrared spectra of histamine complexes with palladium(II). Vib. Spectrosc..

[35] Xerri B., Flament J.-P., Petitjean H., Berthomieu C., Berthomieu D. (2009). Vibrational modeling of copper-histamine complexes: Metal-ligand IR modes investigation. J. Phys. Chem. B.

[36] Homeyer N., Gohlke H. (2012). Free energy calculations by the molecular mechanics Poisson−Boltzmann surface area method. Mol. Inform..

[37] Birdsall N.J. (1991). Cloning and structure-function of the H_2_ histamine receptor. Trends Pharmacol. Sci..

[38] Gantz I., DelValle J., Wang L.D., Tashiro T., Munzert G., Guo Y.J., Konda Y., Yamada T. (1992). Molecular basis for the interaction of histamine with the histamine H_2_ receptor. J. Biol. Chem..

[39] Ramesh Kumara G., Gokul Raj S., Saxena A., Karnal A.K., Raghavalu T., Mohan R. (2008). Deuteration effects on structural, thermal, linear and nonlinear properties of L-threonine single crystals. Mater. Chem. Phys..

[40] Zhang J., Qi T., Wei J. (2012). Homology Modeling and Antagonist Binding Site Study of the Human Histamine H2 Receptor. Med. Chem..

[41] Strasser A. (2009). Molecular modeling and QSAR-based design of histamine receptor ligands. Expert Opin. Drug Discov..

[42] Catalan J., Abboud J.L.M., Elguero J. (1897). Basicity and Acidity of Azoles. Adv. Heterocycl. Chem..

[43] Frisch M.J., Trucks G.W., Schlegel H.B., Scuseria G.E., Robb M.A., Cheeseman J.R., Scalmani G., Barone V., Petersson G.A., Nakatsuji H. (2016). Gaussian 16, Revision C.01.

[44] Marenich A.V., Cramer C.J., Truhlar D.G. (2009). Universal Solvation Model Based on Solute Electron Density and on a Continuum Model of the Solvent Defined by the Bulk Dielectric Constant and Atomic Surface Tensions. J. Phys. Chem. B.

[45] Grosdidier A., Zoete V., Michielin O. (2011). SwissDock, a protein-small molecule docking web service based on EADock DSS. Nucleic Acids Res..

[46] Pettersen E.F., Goddard T.D., Huang C.C., Couch G.S., Greenblatt D.M., Meng E.C., Ferrin T.E. (2004). UCSF Chimera—A Visualization System for Exploratory Research and Analysis. J. Comput. Chem..

[47] Case D.A., Betz R.M., Cerutti D.S., Cheatham T.E., Darden T.A., Duke R.E., Giese T.J., Gohlke H., Goetz A.W., Homeyer N. (2016). Amber 2016.

[48] Darden T., York D., Pedersen L. (1993). Particle Mesh Ewald: An *N*∙log(*N*) Method for Ewald Sums in Large Systems. J. Chem. Phys..

[49] Genheden S., Ryde U. (2015). The MM/PBSA and MM/GBSA Methods to Estimate Ligand-Binding Affinities. Expert Opin. Drug Discov..

[50] Hou T., Wang J., Li Y., Wang W. (2011). Assessing the Performance of the MM/PBSA and MM/GBSA Methods. 1. The Accuracy of Binding Free Energy Calculations Based on Molecular Dynamics Simulations. J. Chem. Inf. Model..

[51] Tandarić T., Vianello R. (2019). Computational Insight into the Mechanism of the Irreversible Inhibition of Monoamine Oxidase Enzymes by the Antiparkinsonian Propargylamine Inhibitors Rasagiline and Selegiline. ACS Chem. Neurosci..

[52] Perković I., Raić-Malić S., Fontinha D., Prudêncio M., Pessanha de Carvalho L., Held J., Tandarić T., Vianello R., Zorc B., Rajić Z. (2020). Harmicines—Harmine and Cinnamic Acid Hybrids as Novel Antiplasmodial Hits. Eur. J. Med. Chem..

[53] Gohlke H., Kiel C., Case D.A. (2003). Insights into Protein-Protein Binding by Binding Free Energy Calculation and Free Energy Decomposition for the Ras-Raf and Ras-RalGDS Complexes. J. Mol. Biol..

[54] Rastelli G., Del Rio A., Degliesposti G., Sgobba M. (2010). Fast and Accurate Predictions of Binding Free Energies Using MM-PBSA and MM-GBSA. J. Comput. Chem..

[55] Olsson M.H.M., Søndergaard C.R., Rostkowski M., Jensen J.H. (2011). PROPKA3: Consistent treatment of internal and surface residues in empirical p*K*_a_ predictions. J. Chem. Theory Comput..

[56] Liao R.Z., Georgieva P., Yu J.G., Himo F. (2011). Mechanism of mycolic acid cyclopropane synthase: A theoretical study. Biochemistry.

[57] Vianello R., Repič M., Mavri J. (2012). How are biogenic amines metabolized by monoamine oxidases?. Eur. J. Org. Chem..

[58] Maršavelski A., Vianello R. (2017). What a difference a methyl group makes: The selectivity of monoamine oxidase B towards histamine and *N*-methylhistamine. Chem. Eur. J..

[59] Himo F. (2017). Recent trends in quantum chemical modeling of enzymatic reactions. J. Am. Chem. Soc..

[60] Blomberg M.R.A., Borowski T., Himo F., Liao R.-Z., Siegbahn P.E.M. (2014). Quantum chemical studies of mechanisms for metalloenzymes. Chem. Rev..

[61] Quesne M.G., Borowski T., de Visser S.P. (2016). Quantum mechanics/molecular mechanics modeling of enzymatic processes: Caveats and breakthroughs. Chem. Eur. J..

[62] Sousa S.F., Ribeiro A.J.M., Neves R.P.P., Brás N.F., Cerqueira N.M.F.S.A., Fernandes P.A., Ramos M.J. (2017). Application of quantum mechanics/molecular mechanics methods in the study of enzymatic reaction mechanisms. Wires Comput. Mol. Sci..

[63] Quesne M.G., Silveri F., de Leeuw N.H., Catlow C.R.A. (2019). Advances in sustainable catalysis: A computational perspective. Front. Chem..

[64] Kaur S., Gupta M. (2017). Deuteration as a tool for optimization of metabolic stability and toxicity of drugs. Glob. J. Pharm. Sci..

[65] Tung R.D. (2016). Deuterium medicinal chemistry comes of age. Future Med. Chem..

[66] Don C.G., Riniker S. (2014). Scents and sense: In silico perspectives on olfactory receptors. J. Comput. Chem..

