# Supplementary material for: The Effect of Deuteration on the H2 Receptor Histamine Binding Profile: A Computational Insight into Modified Hydrogen Bonding Interactions
Source: Molecules. 2020 Dec 18;25(24):6017. doi: 10.3390/molecules25246017 (PMC7766521; doi:10.3390/molecules25246017)
Supplement: Supplementary file 1 [file molecules-25-06017-s001.pdf]

SUPPLEMENTARY MATERIALS

The effect of deuteration on the histamine binding profile towards H_2 receptor: A computational insight into the modified hydrogen bonding interactions

Lucija Hok [1], Janez Mavri [2] and Robert Vianello [1,*]

[1] Division of Organic Chemistry and Biochemistry, Ruđer Bošković Institute, Zagreb, Croatia; lucija.hok@irb.hr (L.H.); robert.vianello@irb.hr (R.V.)

[2] Laboratory for Computational Biochemistry and Drug Design, National Institute of Chemistry, Ljubljana, Slovenia; janez.mavri@ki.si (J.M.)

* Correspondence: robert.vianello@irb.hr

CONTENT	PAGE
**Figure S1.** Time dependence of N1–N2 distances (left) and dihedral angles describing the rotation of the free ethylamino group around the imidazole ring (right) during the molecular dynamics simulation of histamine monocation in aqueous solution.	S2
**Figure S2.** RMSD graphs during the molecular dynamics simulation of the H_2 receptor without the ligand (left) and with the bound histamine monocation (right).	S3
**Figure S3.** Time dependence of N2(histamine)···**C**OO(Asp98) distances describing the N–H···O hydrogen bonding between the protonated amino group on histamine and the side chain carboxyl group in Asp98 (left), and the corresponding number of the formed hydrogen bonding contacts (right) during the molecular dynamics simulation.	S4
**Figure S4.** Time dependence of N2(histamine)···O(Tyr250) distances (top left) and the corresponding number of the formed hydrogen bonding contacts (top right), and the evolution of O(Tyr250)···**C**OO(Asp98) distances (bottom left) and the matching number of the formed hydrogen bonding contacts (bottom right) during the molecular dynamics simulation.	S5
**Figure S5.** The evolution of N3(histamine)···O(Thr103) distances (top left) and the corresponding number of the formed hydrogen bonding contacts (top right), and the evolution of N3(histamine)···O(Thr190) distances (bottom left) and the matching number of the formed hydrogen bonding contacts (bottom right) during the molecular dynamics simulation.	S6
**Figure S6.** The evolution of distances between the hydroxyl O-atom in the side chain –OH group of Thr190 and the backbone carbonyl O-atom of Asp186 (top left) and the matching number of the formed hydrogen bonding contacts (top right) during the molecular dynamics simulation. A representative snapshot describing this interaction is depicted in bottom.	S7
**Figure S7.** Less favorable histamine orientation within the H_2 receptor binding site, which is associated with an increased individual contribution from Thr190 and Asp186 residues, yet the overall histamine binding affinity is 3.3 kcal mol^{-1} less exergonic than for the most favorable binding discussed in the text.	S8
**Figure S8.** Distribution of the relevant N1–N2 distances (left) and dihedral angles describing the rotation of the free ethylamino group around the imidazole ring (right) during the molecular dynamics simulation of histamine bound within the H_2 receptor.	S9

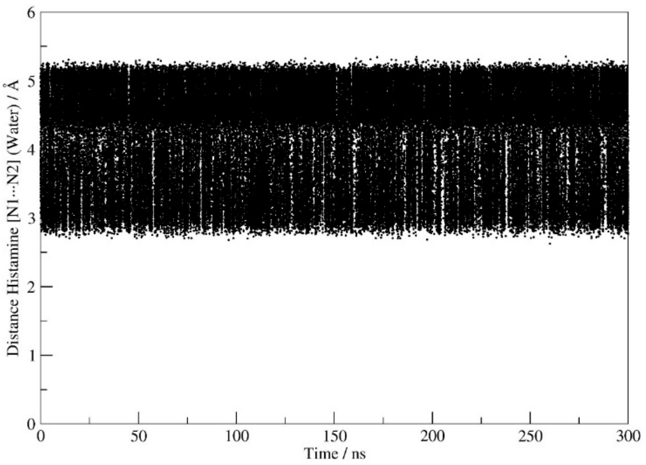

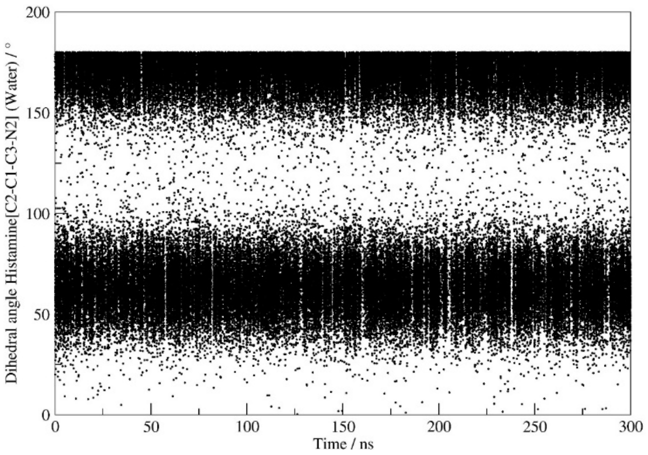

Figure S1. Time dependence of N1–N2 distances (left) and dihedral angles describing the rotation of the free ethylamino group around the imidazole ring (right) during the molecular dynamics simulation of histamine monocation in aqueous solution.

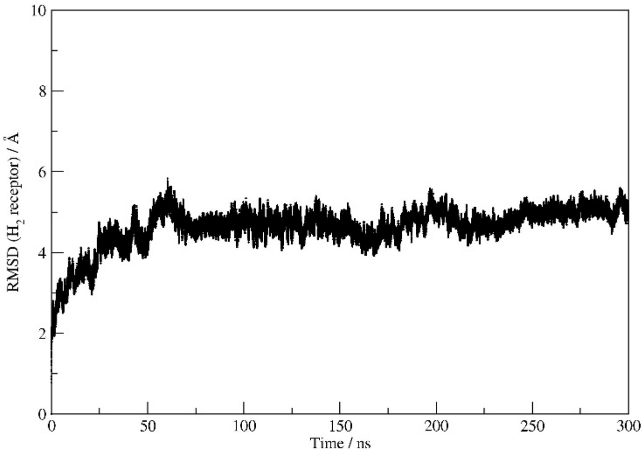

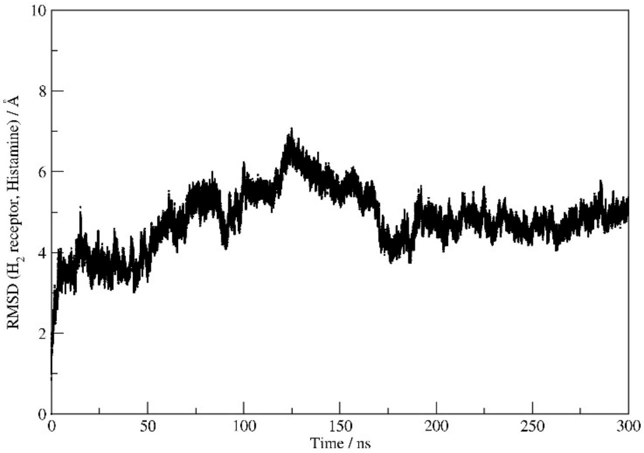

Figure S2. RMSD graphs during the molecular dynamics simulation of the H_2 receptor without the ligand (left) and with the bound histamine monocation (right).

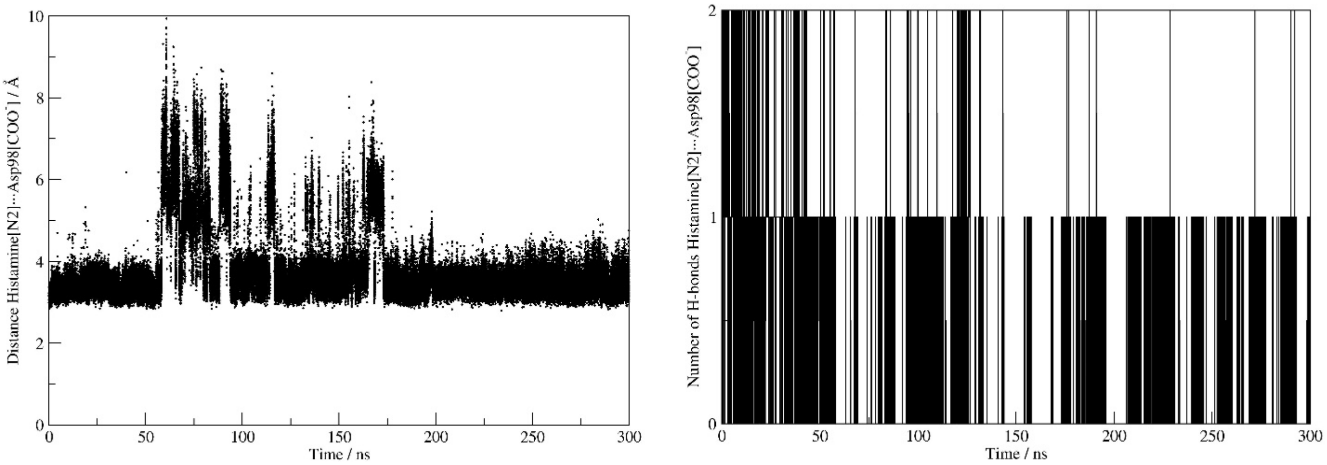

Figure S3. Time dependence of N2(histamine)···**C**OO(Asp98) distances describing the N–H···O hydrogen bonding between the protonated amino group on histamine and the side chain carboxyl group in Asp98 (left), and the corresponding number of the formed hydrogen bonding contacts (right) during the molecular dynamics simulation.

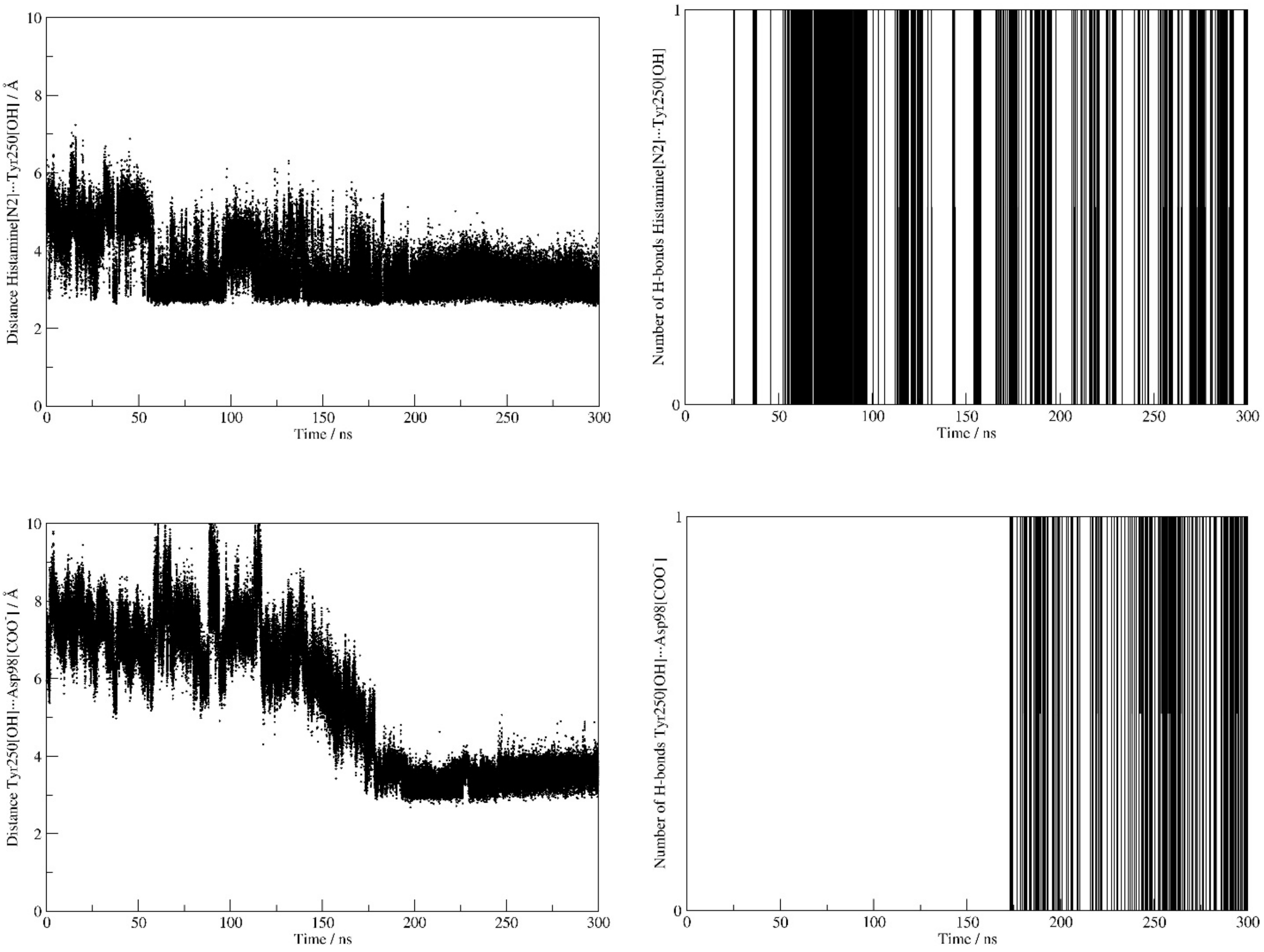

Figure S4. Time dependence of N2(histamine)···O(Tyr250) distances (top left) and the corresponding number of the formed hydrogen bonding contacts (top right), and the evolution of O(Tyr250)···**C**OO(Asp98) distances (bottom left) and the matching number of the formed hydrogen bonding contacts (bottom right) during the molecular dynamics simulation.

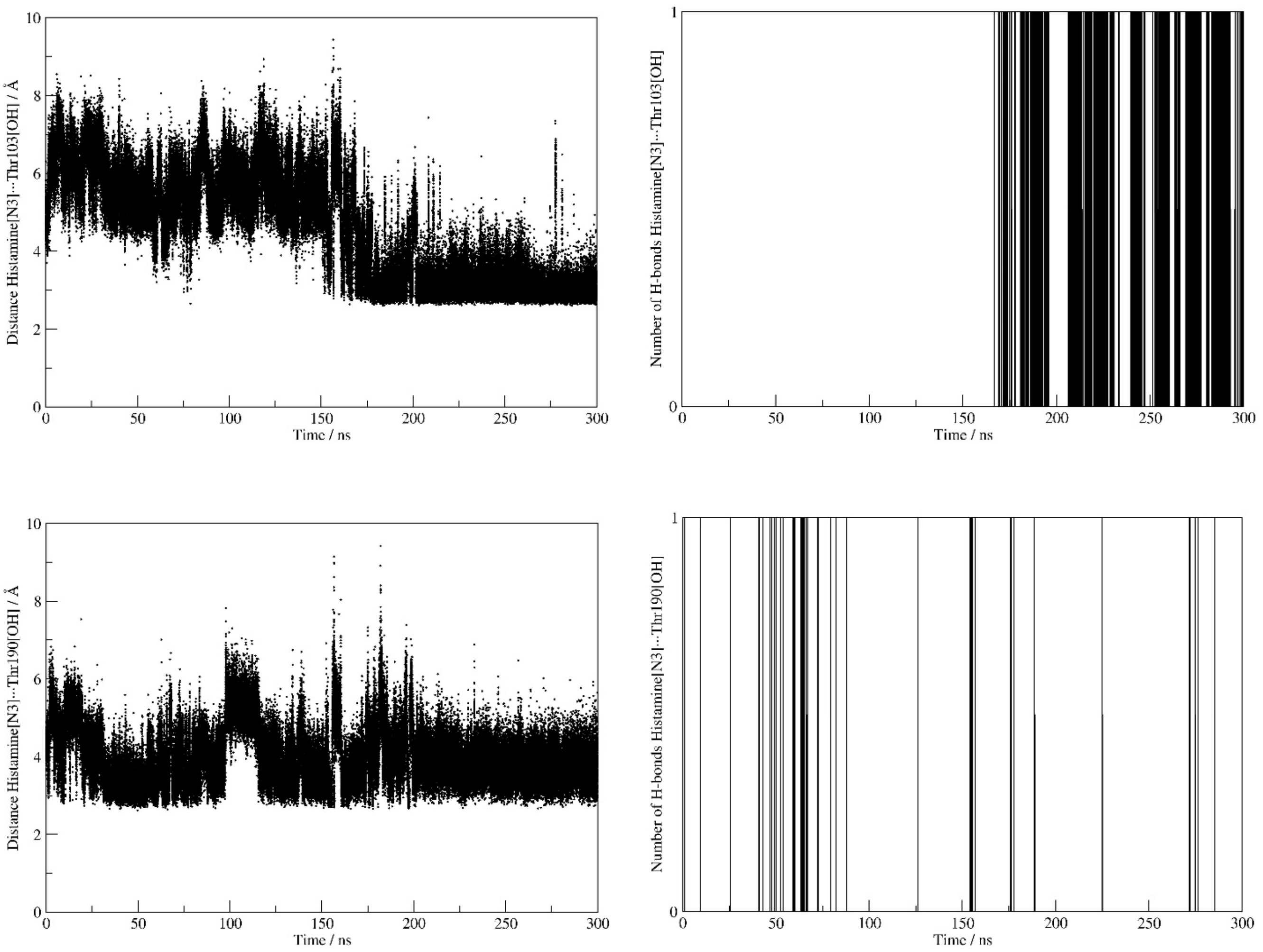

Figure S5. The evolution of N3(histamine)···O(Thr103) distances (top left) and the corresponding number of the formed hydrogen bonding contacts (top right), and the evolution of N3(histamine)···O(Thr190) distances (bottom left) and the matching number of the formed hydrogen bonding contacts (bottom right) during the molecular dynamics simulation.

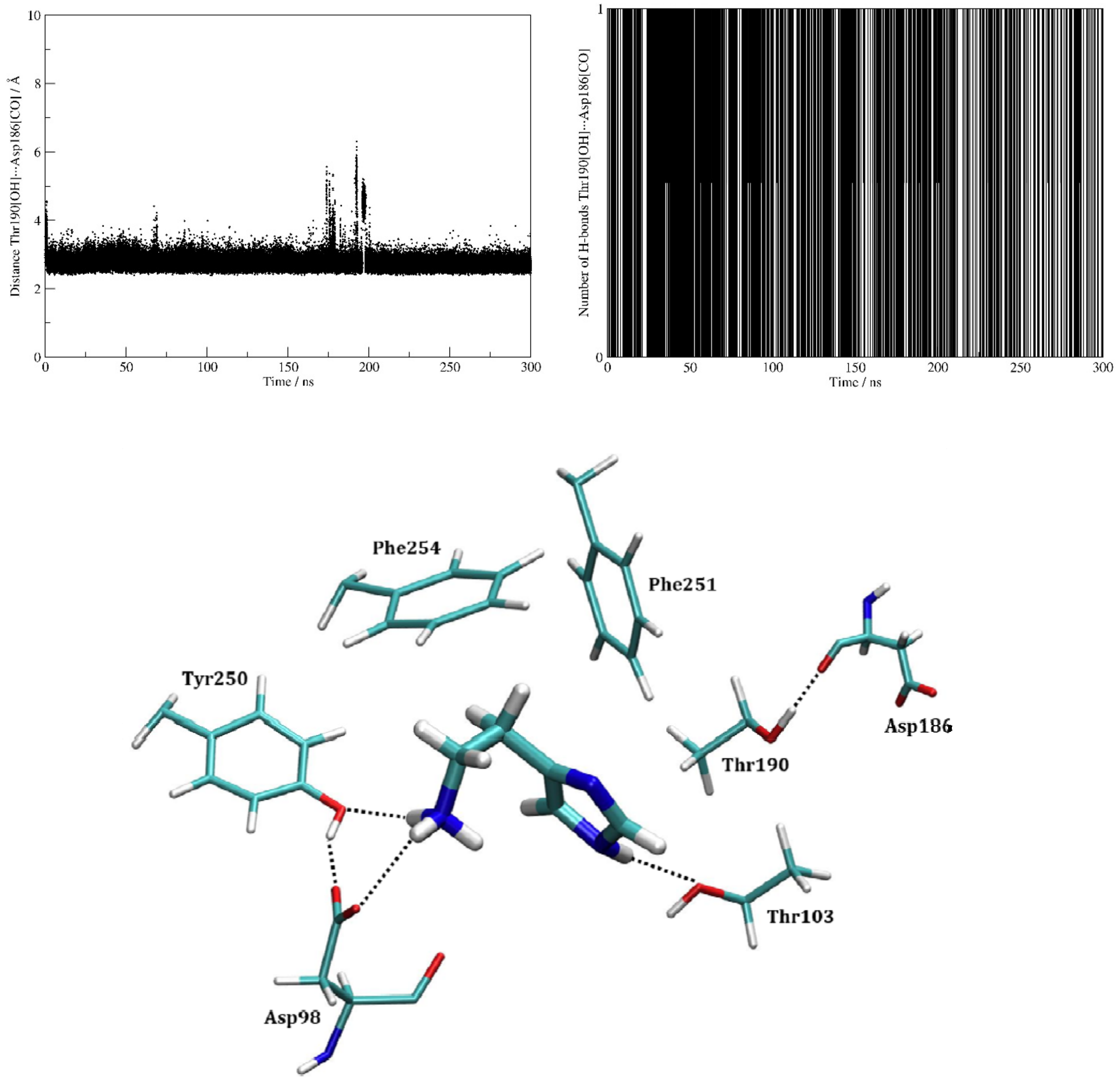

Figure S6. The evolution of distances between the hydroxyl O-atom in the side chain –OH group of Thr190 and the backbone carbonyl O-atom of Asp186 (top left) and the matching number of the formed hydrogen bonding contacts (top right) during the molecular dynamics simulation. A representative snapshot describing this interaction is depicted in bottom.

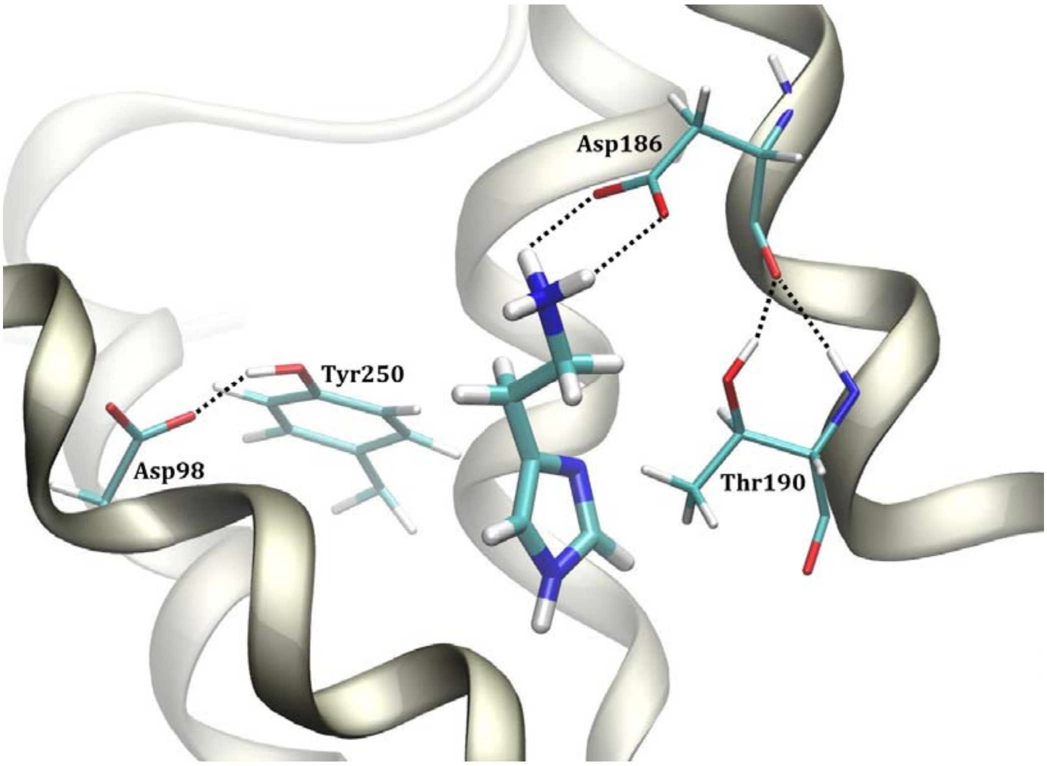

Figure S7. Less favorable histamine orientation within the H_2 receptor binding site, which is associated with an increased individual contribution from Thr190 and Asp186 residues, yet the overall histamine binding affinity is 3.3 kcal mol^{-1} less exergonic than for the most favorable binding discussed in the text.

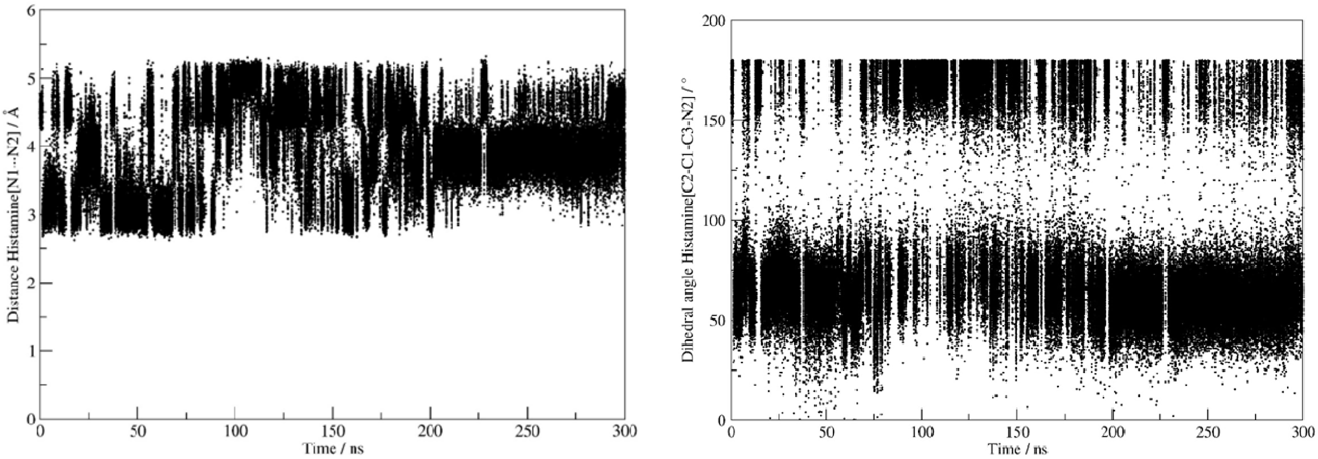

Figure S8. Distribution of the relevant N1–N2 distances (left) and dihedral angles describing the rotation of the free ethylamino group around the imidazole ring (right) during the molecular dynamics simulation of histamine bound within the H_2 receptor.
